# Sex differences in potassium regulation: evidence, molecular mechanisms and clinical implications

**DOI:** 10.1186/s13293-026-00862-4

**Published:** 2026-03-01

**Authors:** Lama Al-Qusairi

**Affiliations:** Division of Nephrology, Johns Hopkins School of Medicine, 720 Rutland Ave, Baltimore, 21205 MD USA

## Abstract

Men and women exhibit well-established differences in electrolyte regulation, susceptibility to hypertension, kidney disease prevalence, and response to therapies for cardiovascular and renal disorders. Among these, plasma potassium regulation emerges as a key area of sex-specific divergence. Clinical and experimental studies consistently show that women are more susceptible to hypokalemia, while men are more prone to hyperkalemia, a pattern most evident during physiological or pathological stressors such as specific dietary patterns, exercise, medication use, and diseases like chronic kidney disease or cardiorenal syndrome. In this review, we synthesize current evidence from both human and animal studies on sex differences in potassium homeostasis and discuss their clinical implications. We also explore the physiological and molecular mechanisms underlying these differences, and highlight critical knowledge gaps. Advancing our understanding of these biological differences and their underlying mechanisms is essential to move beyond one-size-fits-all nutritional recommendations and disease management strategies.

**Graphical Abstract:**

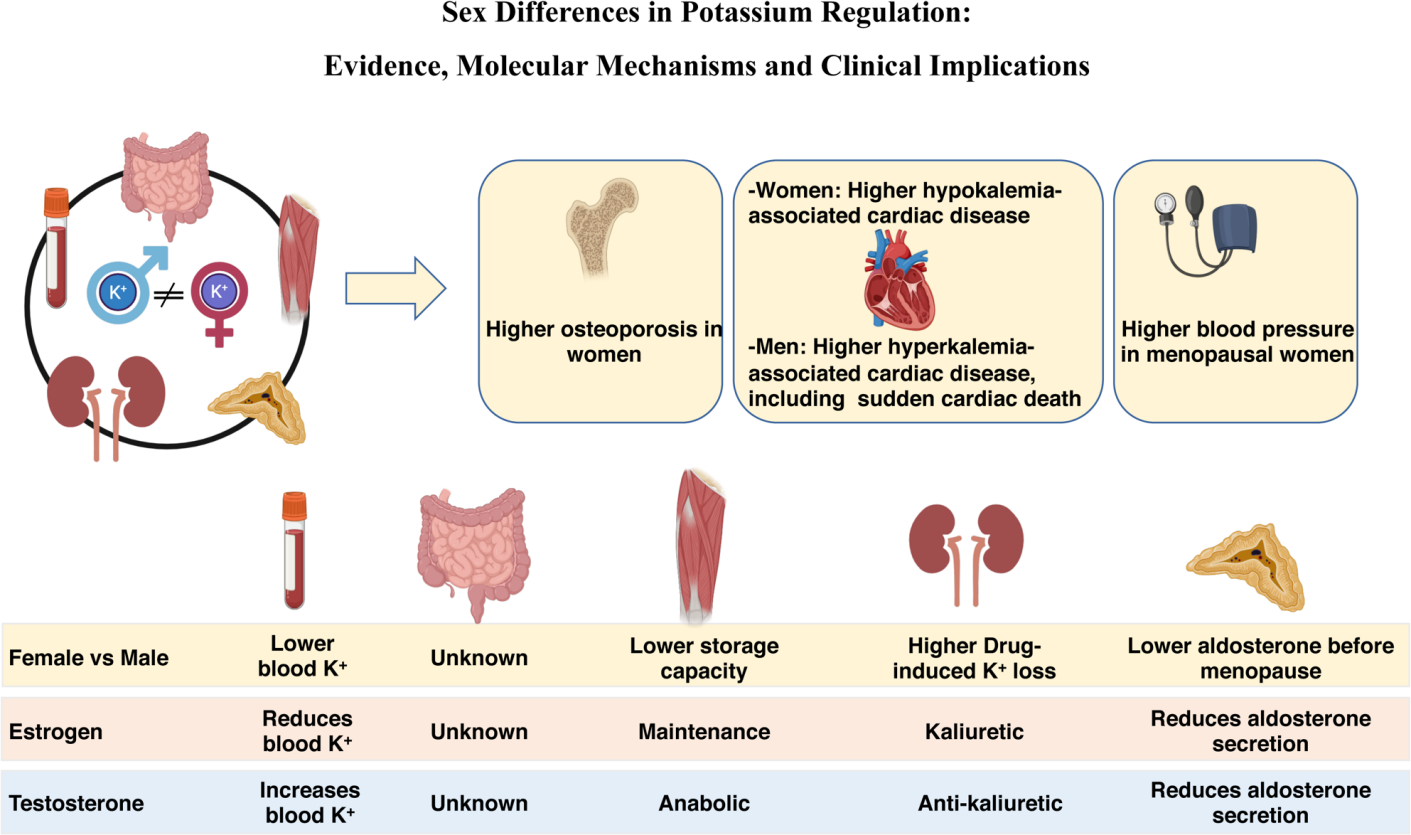

## Introduction

Potassium (Kalium: K^+^), one of the most abundant cations in the body, is tightly regulated to maintain a steep concentration gradient, approximately 140 mM intracellular and 3.5–5 mM extracellular (plasma) [1, 2]. This gradient is essential for establishing the resting membrane potential, enabling cellular excitability and responsiveness to stimuli. Beyond its electrical role, intracellular K⁺ is also critical for regulating cell volume, proliferation, and apoptosis [3, 4]. Throughout life, the K⁺ homeostatic system is continuously challenged by physiological demands and environmental stressors, either acutely, such as during meals and exercise, or chronically, during diseases, long-term dietary variations, pregnancy, and lactation. Because even slight deviations in plasma K⁺ can have life-threatening consequences, regulatory mechanisms are highly sensitive and precisely tuned. When these mechanisms are overwhelmed, disturbances in K⁺ levels can result, leading to significant clinical consequences. Both hypokalemia and hyperkalemia are associated with increased morbidity and mortality, observed across a range of conditions, including chronic kidney disease [5], cardiovascular disorders [6, 7], emergency care admissions [8], and even in the general population [9].

It is anticipated that K^+^ homeostasis is handled differently in men and women, especially in the broader context of sex-based physiological differences in renal [10–17], hormonal [18, 19] and muscular systems [20]. Large-scale epidemiological studies have consistently shown sex differences in basal plasma K^+^ levels that are amplified by physiological and pathophysiological challenges [21–23]. Despite these insights, the mechanisms underpinning sex-based K^+^ regulation remain poorly defined, and current clinical guidelines of K^+^ management often overlook sex as a relevant biological variable. Understanding the physiological and molecular mechanisms underlying sex differences in K^+^ metabolism is essential for tailoring medical practices and dietary guidelines to meet the distinct needs of men and women. Currently, the American National Institute of Health (NIH) dietary recommendations suggest a daily K^+^ intake of 3,400 mg for men and 2,600 mg for women, guidelines based primarily on body mass rather than physiological or molecular insights into K^+^ homeostasis. Notably, female patients with Gitelman syndrome, an inherited hypokalemic alkalosis, involving urinary K^+^ loss [24, 25], require higher K^+^ supplementation than male patients [26], further emphasizing the need for sex-specific approaches to K^+^ management.

This review integrates epidemiological evidence on sex differences in K^+^ homeostasis with multi-system physiological and molecular findings that explain, or at least support, these differences. We further attempt to connect these differences to their clinical relevance. Overall, this work provides a comprehensive perspective intended to inform basic and translational research and patient care. We will not cover the regulation of K^+^ homeostasis during pregnancy, which has been discussed previously [27].

## Sex differences in basal plasma potassium and dyskalemia risk

### Basal plasma K⁺ in the general population

Population-based analyses from the US National Health and Nutrition Examination Surveys (NHANES) showed women exhibit lower basal plasma K^+^ levels than men by approximately 0.15 mmol/L across all age groups Table 1 [21, 22]. These data are consistent with a population study from China showing plasma K^+^ concentration was lower in women than men Table 1 [23]. Although studies in pediatric populations are limited, available evidence suggests that sex differences in plasma K^+^ levels do not emerge before puberty. In a study of healthy Han children in China, no significant sex differences in plasma K⁺ levels were observed Table 1 [28]. Similarly, a 24-h urinary K^+^ excretion study in a Portuguese pediatric cohort found no sex-based differences Table 1 [29]. Together, sex differences in basal K^+^ appear with puberty and persist after menopause and andropause.

**Table 1:** Epidemiological and clinical studies addressing plasma K + or urinary K + excretion in healthy or diseased populations

Study name (Ref #)	Country	sample size	Women (%)	age	major findings
SCREAM [30]	Sweden	364,955	56	≥ 18y	More hypokalemia in women, more hyperkalemia in men
Einhorn,s [38]	USA	245,808	4.4	18y - >76y	Men had 2X higher hyperkalemia
Maida’s [45]	Japan	67135	52	∼ 75	More hypokalemia in women, more hyperkalemia in men upon diuretics use
NHANES [21]	USA	57,996	51	12y - 80y	Women have lower basal blood K+, and 2X more hypokalemia
Wang’s [23]	China	43,151	45	11y-100y	Women have lower basal K+
NHANES [22]	USA	18,723	52	≥ 12y	Women have lower blood plasma K+, and 2.7X more hypokalemia
Takaichi’s [39]	Japan	9,196	42	∼ 60	Male sex as an independent risk factor for hyperkalemia
Rotterdam [31]	Netherlands	4,059	60	≥ 55y	3x more hypokalemia in women
NEFRONA [40]	Spain	2,891	39	18y-75y	Hyperkalemia is more common and more severe in men
Zhou’s [28]	China	1,391	46	2y - 15y	No difference in plasma K+ in childhood
Jin’s [43]	China	1,266	18	≥ 55y	Higher serum K^+^ levels were associated with male sex
Cardioren [41]	Spain	1,107	37	64y –82y	In cardioenal patients: Hyperkalemia is more common in men
Kleinfeld’s [32]	USA	872	59	≥ 65	3x more hypokalemia in women
Korgaonkar’s [42]	USA	820	45	45y-76y	In CKD patients: 3X more hyperkalemia in men
Sever’s [44]	Turkey	639	46	16y-47y	Rhabdomyolysis-induced hyperkalemia is higher in men
Toner’s [35]	USA	193	53	∼ 55	Thiazide-induced hypokalemia is more comon in women
Oliveira’s [29]	Portugal	163	50	8y - 10y	No difference in 24h K+ excretion in childhood
Clark’s [36]	USA	161	79	64y-96y	Diuretics-induced hypokalemia is 5X higher in women
van der Heijden's [37]	Netherlands	160	54	∼ 70	Penicillin-like antibiotics-induced hypokalemia is 2X higher in women

### Women are more prone to hypokalemia

Several population studies in the US and Europe have shown higher susceptibility of women to hypokalemia. US NHANES-based studies have revealed women were 2.5 times more likely to develop hypokalemia Table 1 [21, 22]. In the observational Stockholm CREAtinine Measurements (SCREAM) project, women had a significantly higher risk of hypokalemia Table 1 [30]. Similarly, in the prospective Rotterdam Study, hypokalemia incidence was threefold higher in women than in men Table 1 [31]. Studies in hospitalized patients are consistent with findings from the general population. Indeed, among 872 hospitalized adults, women over 65 years had a threefold greater risk of hypokalemia compared to age-matched men Table 1 [32]. In another independent cohort of 73 hospitalized patients with profound hypokalemia, women represented the majority of cases Table 1 [33].

Sex-specific differences in hypokalemia risk have been also described in Medication-Induced Hypokalemia. Various medications contribute to hypokalemia, including diuretics, which enhance distal Na^+^ delivery and hence K^+^ secretion, and β-lactam antibiotics, which act as non-reabsorbable anions promoting renal K^+^ loss [34]. In a cohort of 193 hypertensive patients treated with thiazides, women had lower baseline plasma K^+^ and twice the risk of hypokalemia compared to men Table 1 [35]. The sex difference seems to be higher in the elderly population, as shown by a study of 161 elderly nursing-home residents on diuretics, with women having five times higher incidence than age-matched men Table 1 [36]. Similarly, among over 160 patients receiving penicillin-like (containing β-lactam ring) antibiotics, 14–44% developed hypokalemia depending on dose, with women facing twice the risk compared to men Table 1 [37]. However, it remains unclear whether female sex influences medication-induced hypokalemia in children. Given the frequent use of antibiotics in pediatric populations, a sex-stratified analysis of the effects of β-lactams on K^+^ balance is still awaiting.

### Men are more prone to hyperkalemia

A retrospective analysis of over two million records from nearly 246,000 US veterans revealed that men had a two-fold higher risk of developing hyperkalemia compared to women Table 1 [38]. Data from the European SCREAM project and a large cohort of kidney disease patients in Japan identified male sex as an independent risk factor for hyperkalemia Table 1 [30, 39]. The NEFRONA study from Spain, showed that serum K^+^ levels, as well as the prevalence of hyperkalemia and severe hyperkalemia, were significantly higher in men than in women with CKD stages G3–G5 Table 1 [40]. Likewise, data from the Spanish CARDIOREN registry indicated a greater incidence of hyperkalemia among male patients Table 1 [41]. In a US-based CKD cohort study involving 820 participants, 77% of all hyperkalemia cases occurred in men Table 1 [42]. In a community-based study of elderly Chinese residents, higher serum K^+^ levels were significantly associated with age and male sex Table 1 [43]. In a cohort of 639 victims of crush syndrome in Turkey, the rhabdomyolysis-induced hyperkalemia was higher in men than women at admission Table 1 [44].

Additionally, drug-induced hyperkalemia is higher in men than in women. In a cohort regrouping 67,135 patients on diuretics in Japan, men were found to be more prone to hyperkalemia, while women were more prone to hypokalemia Table 1 [45].

Together, these reports indicate that female susceptibility to hypokalemia and male susceptibility to hyperkalemia are consistent across diverse populations worldwide, suggesting these sex differences are independent of geographic or ethnic variation.

## Mechanisms underlying the K^+^-related sex differences

Consistent with findings in humans, data from mice [12, 46, 47] and rats [48, 49] confirm a sex difference in basal plasma K^+^, and show that females are more susceptible to hypokalemia [46, 47, 49] while males are more susceptible to hyperkalemia [49–53]. This sex-specific susceptibility becomes evident under challenging conditions, such as dietary K^+^ manipulations [46, 47, 49]; or in disease models of rhabdomyolysis [49], and altered renal K^+^ transport [50–53]. This indicates a conserved mechanism in mammals and offers an opportunity to employ rodents as adequate models to study the physiological and molecular mechanisms underlying these sex differences. In terrestrial mammals, K^+^ homeostasis is maintained through a complex coordination of multiple organs: intestinal absorption and secretion in the intestine, skeletal muscle buffering, and renal excretion, processes further modulated by hormonal signals from the insulin-producing pancreas and the aldosterone-secreting adrenal glands (see review [2]). In this section, we synthesize findings from clinical and animal studies to shed light on the biological basis of these sex-specific differences.

### Sex-specific regulation of skeletal muscle K^+^ buffering

After a K^+^-rich meal, the absorbed K^+^ is rapidly buffered by the body’s intracellular K^+^ stores, mainly skeletal muscle and, to a lesser extent, the liver, red blood cells, and bones [1]. Skeletal muscle, which contains over 75% of total body K^+^, serves as a critical buffer during both acute fluctuations (e.g., exercise or meals) [54] and chronic alterations (e.g., K^+^ disorders or long-term dietary variations) [55]. On average, men have over 30% more skeletal muscle mass than women [56], primarily due to the anabolic and anti-catabolic effects of testosterone, which promotes muscle protein synthesis and reduces degradation [57–61]. In contrast, estrogen exerts mainly protective and maintenance roles on female skeletal muscle, as shown during post-injury recovery or in postmenopausal hormonal therapy [62–65]. One may expect that this larger K^+^ reservoir in men provides greater protection against hypokalemia, but prone them to hyperkalemia upon physiological challenge. This assumption is supported by observations in victims of rhabdomyolysis and exercise-related sudden cardiac death, two conditions associated with K^+^ release from skeletal muscle to the bloodstream, which are more severe in men [44, 50, 66–68]. Animal studies confirm these conclusions, showing that rhabdomyolysis results in higher plasma K^+^ and higher mortality rate in male compared to female mice [50]. However, direct evidence linking skeletal muscle mass to women’s susceptibility to hypokalemia and men’s susceptibility to hyperkalemia remains to be established.

The classical view of K^+^ homeostasis assumes tightly maintained intracellular K^+^ levels (~ 140 mM), yet sex differences in intracellular K^+^ content remain poorly defined. Male rodents show higher basal K^+^ in skeletal muscle [69] and spleen [70]. Mouse studies have demonstrated that prolonged dietary K^+^ deficiency (30 days) induces sex-specific reductions in intracellular K^+^ content. Indeed, while tissue K^+^ content in the pancreas and skeletal muscle decline in both sexes, reductions in kidney, liver, and heart tissue K^+^ content are observed exclusively in females [69]. K^+^ buffering in skeletal muscle occurs via a tightly regulated balance between K^+^ efflux and uptake within the myofiber. In the postprandial phase upon a K^+^ rich meal (1-6 h), plasma K^+^ increases to a high level, reaching 7 mM in animals fed an experimental high K^+^ diet [71, 72]. Half of this K^+^ load is excreted by the kidney, as observed in animals and humans [73, 74], while the other half shifts to the intracellular K^+^ store, mainly in skeletal muscle via Na^+^/K^+^-ATPase, protecting excitable organs against excessive hyperkalemia [2, 55, 75]. Similarly to the effect of a K^+^-rich meal, skeletal muscle contraction involving depolarization activates voltage-gated K^+^ channels, resulting in K^+^ efflux and transient elevations in plasma K^+^, reaching 6–8 mM in humans [76]. This extreme challenge is also overcome by a rapid stimulation of Na^+^/K^+^-ATPase [2, 54, 55], which uptakes K^+^ into skeletal muscle. Studies in a rodent model of testosterone deficiency (castration) revealed that castration reduces skeletal muscle Na^+^/K^+^-ATPase expression, arguing for a positive regulation of the pump by testosterone [20]. It is noteworthy that over 80% of exercise-related sudden cardiac death victims are men, as has been shown in several cohorts from Spain, France, and the US [66–68]. It is still unclear whether the human Na^+^/K^+^-ATPase is regulated by sex hormones, and how skeletal muscle K^+^ efflux is regulated in men and women remains unexplored.

### Sex-specific regulation of renal K^+^ handling

Human studies analyzing the sex difference in K^+^ excretion are limited. Population-based epidemiological surveys conducted in Belgium (N = 8,058; 48% women) and Australia (N = 56; 57% women) showed that men had a 22% and 13% higher K^+^ excretion than women, respectively [77, 78]. Estimation of K^+^ intake suggests that men consume more K^+^ than women; however, the difference was not maintained after adjustment for total caloric intake [79]. In a cross-sectional study from China involving hospitalized hypertensive patients (N = 606; 39% women), K^+^ excretion was similar between sexes [80], but information on K^+^ intake was not available. Measurement of 24 h urinary K^+^ excretion in Angiotensin II (Ang II)-rats, revealed higher urinary K^+^ excretion in females than males, when excretion was normalized by body weight [81].

Renal K^+^ regulation (for reviews [2, 82, 83]) is the sum of multiple segment-specific K^+^ handling mechanisms. After K^+^ is freely filtered by the glomerulus, 90% of the filtered K^+^ is reabsorbed by passive paracellular pathway, mainly in the proximal tubules (PT) (70–80%) and to a lesser extent in the thick ascending limb (TAL) [2]. The fine-tuning of K^+^ excretion occurs in the distal nephron, which extends from the late part of the distal convoluted Tubules (DCT2), to the connecting tubules (CNT) and the collecting duct (CD), where K^+^ is secreted into the pro-urine via both electrogenic and electroneutral mechanisms [2, 84–86]. Electrogenic K^+^ secretion is primarily driven by the epithelial sodium channel (ENaC), located at the apical membrane of principal cells, which reabsorbs Na^+^, creating by that a favorable lumen-negative potential to facilitate K^+^ secretion. In this system, K^+^ exit is mediated by the renal outer medullary K^+^ channels (ROMK), operating in the principal cells; and the flow-dependent Big-K^+^ channel (BK), operating in the principal and intercalated cells of the distal nephron [2, 87]. K^+^-mediated ENaC activation is tightly regulated by upstream sodium delivery, defined by the action of the thiazide-sensitive sodium-chloride cotransporter (NCC) in the DCT. NCC action is regulated by extracellular K^+^ via the basolateral K^+^ channels, Kir4.1/5.1 that acts as a K^+^ sensor [88–90]. It allows small physiological changes in blood K^+^[71, 91–93] to signal to a specific network of WNK-SPAK kinases [94] and K^+^-regulated phosphatases [92] that directly regulates NCC activity. This signaling, coined the K^+^ switch, allows NCC-mediated distal Na^+^ delivery to be directly and tightly regulated by plasma K^+^, where low K^+^ reduces Na^+^ delivery and high K^+^ enhances Na^+^ delivery to achieve ENaC-mediated K^+^ secretion [71, 91–93]. allowing distal Na^+^ delivery to be directly and tightly regulated by plasma K^+^, where low K^+^ reduces Na^+^ delivery and high K^+^ enhances Na^+^ delivery to achieve ENaC-mediated K^+^ secretion [71, 91–93]. On the other hand, the electroneutral K^+^ secretion, ENaC-independent, might be mediated by the recently identified K^+^ and Cl^−^ cotransporter (KCC3a) [95, 96]. Additionally, under extreme K^+^ requirements, such as during pregnancy, a K^+^-rescue mechanism occurs in the distal nephron, by activating H^+^/K^+^-ATPase, which reabsorbs K^+^ in exchange for H^+^[97, 98].

The kidney is a major target of gonadal hormones, as evidenced by the presence of sex hormone receptors in all nephron segments [99–102]. The role of gonadal hormones in K^+^ regulation was established by studies from humans and animals. Testosterone treatment in both humans and rodents significantly increases total body K^+^ and elevates plasma K^+^ levels  [103, 104]. This effect involves kidney function, as castration in rodents leads to urinary K^+^ loss  [104, 105]. Conversely, ovariectomized female rats develop hyperkalemia due to reduced fractional K^+^ excretion, an effect that can be reversed by treatment with 17β-estradiol (**E2**), but not progesterone [48, 106, 107]. These observations indicate that estradiol promotes kaliuresis while testosterone exerts an anti-kaliuretic effect. Testosterone action is mediated by the androgen receptor (AR), while E2 activates several receptors, including the canonical estradiol receptors ERα and ERβ, or the plasma membrane G-protein coupled estrogen receptor R1 (GPER1). Indeed, pharmacologic treatment with ERα, ERβ or GPER1 agonists induces hypokalemia [48, 108]. Studies in mice have shown that AR expression is restricted to the proximal tubules [99, 102, 109], suggesting that its role in K^+^ excretion might be mediated by controlling distal Na^+^ delivery to the K^+^-secreting segments. Interestingly, mice with kidney-specific AR knock-out (AR-KO) exhibit normal plasma K^+^ under basal conditions [109], however, how AR deletion affects K^+^ balance under challenging conditions remains to be explored. On the other hand, single-cell mRNA profiling indicates E2 receptors are present in several cell types along the proximal and distal nephron. Still, their role in K^+^ regulation remains to be identified [99, 110].

**Fig. 1:**
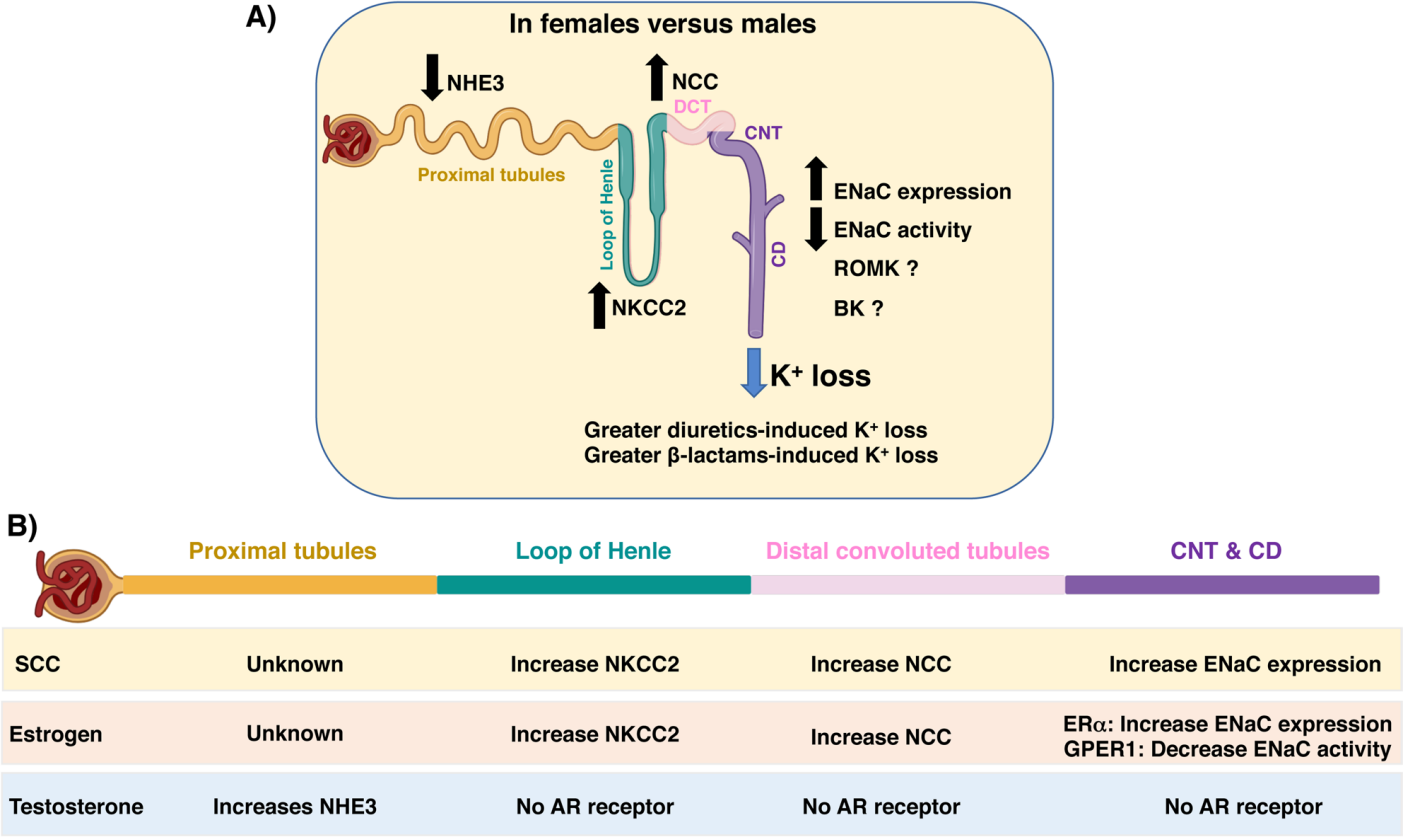
HYPERLINK "sps:id::fig1||locator::gr1||MediaObject::0" Sex differences in renal K⁺ handling (**A**) Schematic representation of the major renal transporters involved in K⁺ excretion and their relative abundance in females compared with males. (**B**) Schematic illustrating the influence of sex chromosome complement (SCC) and gonadal hormones on nephron segments. DCT, distal convoluted tubule; CNT, connecting tubule; CD, collecting duct

Studies in rodents showed that the major Na^+^-reabsorbing pathway in the PT, mediated by the Na^+^/H^+^ exchanger (NHE3) and the Na^+^-dependent Pi transporter 2a (NaPi2a), is less active in females, resulting in higher salt delivery to downstream segments [11, 12, 15, 111, 112]. This observation is supported by data from kidney-specific AR-KO mice showing that AR enhances NHE3 expression [109], consistent with testosterone’s role in K^+^ conservation. Contrasting with the lower Na^+^ reabsorption in the female PT, Na^+^ reabsorption mediated by the furosemide-sensitive Na^+^/K^+^/Cl^−^ cotransporter (NKCC2) in the TAL and thiazide-sensitive NCC in the DCT is higher in females [10–17], more likely driven by E2 [16, 113, 114]. These observations explain the higher incidence of diuretic-induced hypokalemia in women (Fig. 1), which is also supported by pharmacological studies in mice revealing that thiazide or furosemide treatment results in stronger natriuresis and kaliuresis in females [10, 13, 14]. While the physiological relevance of shifting higher Na^+^ reabsorption from proximal to distal segments in females remains unclear, it might represent a homeostatic need as it helps K^+^ conservation and compensates for the lower Na^+^-absorptive capacity in the female proximal tubules.

On the other hand, observations in mice and rats showed that ENaC expression is higher in females, likely driven by the combined effects of the sex chromosome complement and E2-mediated activation of the estrogen receptor α (ERα) [12, 15, 110, 115, 116] (Fig. 1). However, ENaC-mediated natriuresis, as assessed by benzamil, was greater in male rats [117], and amiloride treatment induced greater hyperkalemia and a higher mortality risk in male mice [104], indicating probably higher ENaC activity in males. Recent data revealed that GPER1 is co-expressed with ENaC in the distal nephron, where it downregulates ENaC expression and activity [110, 118]. These observations indicate E2-mediated ENaC regulation is receptor-specific and context-dependent. The contribution of Erα and GPER1 to K^+^ secretion awaits investigation.

Sex differences in K^+^ regulation persist later in life when sex hormone levels decline, suggesting that factors beyond sex hormones, namely the sex chromosome complement (**SCC**), contribute to K^+^ homeostasis. However, the observation that the K^+^-related sex differences appear with puberty highlights the requirement of the activational role of the gonadal hormones to shape these differences, which then become irreversible by sexual hormone decline during menopause and andropause. Disentangling the organizational effects of SCC from the activational role of gonadal hormones is crucial for understanding the consequences of hormonal therapies on K^+^ regulation. A Recent study using the Four Core Genotype mouse model has provided valuable insights. This model allows independent assessment of sex chromosome effects (XX vs. XY) and gonadal effects (female vs. male phenotype), as well as interactions between both [119]. The four genotypes are XX gonadal males or females, and XY gonadal males or females, which were generated by crossing XX gonadal females with XY-Sry⁻ gonadal males, in which the testis-determining Sry gene has been deleted from the Y chromosome and inserted onto an autosome [119]. The study showed that the SCC may influence the basal expression of several transporters and channels involved in K^+^ excretion [15]. Related to K^+^ handling, the study confirmed that Na^+^/K^+^/Cl^−^ cotransporter (NKCC2), NCC, and ENaC exhibit higher basal expression in females [15], confirming previous literature [10–17]. Importantly, the study revealed that SCC also influences NCC and ENaC expression, providing the first evidence of SCC and gonadal hormones interaction in kidney physiology [15] (Fig. 1).

Besides, K^+^ exit channels also exhibit sex-specific regulation. Functional studies using KO models of BKα or the mechanosensor Piezo1, involved in BK activation in the intercalated cells, indicated that only males exhibit increased plasma K^+^ [52, 53], suggesting either males are more dependent on intercalated cell BK function, or females have a better ability to compensate for altered BK-mediated K^+^ secretion. Additionally, studies in transgenic mice with altered ROMK and BK membrane trafficking, caused by the deletion of the endocytic receptor ARH (for autosomal recessive hypercholesterolemia), indicated that males and females activate different compensatory mechanisms to stabilize K^+^ homeostasis [120]. Indeed, ARH-KO females exhibited enhanced NCC abundance and phosphorylation, whereas KO males showed reduced ENaC cleavage and diminished BK auxiliary subunits relative to WT mice [120]. However, it remains unknown whether K^+^ exit channels are regulated by SCC or gonadal hormones, and whether they play a role in the observed sex differences in K^+^ homeostasis.

### Sex-specific regulation of aldosterone secretion and tissue responsiveness

Sex differences in K^+^ homeostasis are shaped not only by distinct patterns of gonadal hormones, which directly affect renal, muscular, and, probably, intestinal K^+^ handling, but also by differential regulation of several key hormones involved in K^+^ balance. Among these, the kaliuretic hormone aldosterone plays a central role. Secreted by the zona glomerulosa of the adrenal cortex, aldosterone is primarily stimulated by elevated plasma K^+^ levels and activation of the renin–angiotensin–aldosterone system (RAAS) in response to hypovolemia or hypotension. An increase in plasma K^+^ stimulates aldosterone secretion, which in turn promotes K^+^ excretion primarily through the distal nephron and, to a lesser extent, the colon, helping restore normal plasma K^+^ concentrations.

Premenopausal women exhibit 15–20% lower circulatory aldosterone than age-matched men. Indeed, in healthy individuals (n = 48, mean age 28 years, 50% women), plasma aldosterone levels were significantly lower in women [18]. Similar results were observed in a second cohort (n = 36, mean age 33 years), in which women exhibited lower aldosterone levels on a high-salt diet, with a trend toward lower levels on a low-salt diet compared to diet-matched men [19]. These sex differences persist in hypertensive populations, as in the well-characterized Hypertensive Pathotype cohort (n = 1,592, mean age 42 years, 43% women), women again had lower serum aldosterone levels than men [121]. Similarly, among patients with resistant hypertension (n = 279, 52% women) and normotensive controls (n = 53, 45% women), women exhibited significantly lower plasma and urinary aldosterone levels than men [122]. Additionally, in premenopausal women, aldosterone secretion appears to follow a cyclic pattern: levels rise during the luteal phase, coinciding with elevated progesterone [123–126]. This increase is required to maintain volume and K^+^ homeostasis, compensating for progesterone’s antagonism of the mineralocorticoid receptor [125–127]. Interestingly, this pattern reverses in postmenopausal women, who have higher aldosterone levels than age-matched men, highlighting a potential inhibitory role of estrogen on aldosterone secretion. Indeed, in the Framingham Offspring Study (n = 2,891, 53.2% women, mean age 59 years), aldosterone levels were higher in women than in men [128]. Studies examining sex differences in basal aldosterone levels in rodents have yielded conflicting results reporting higher levels in males [129], in females [121, 130], or no difference between sexes [12, 131], likely due in part to the lack of consideration for estrous cycle stages, or to species-specific regulation.

Despite evidence of sex differences in aldosterone levels, differences in aldosterone response to physiological variations in plasma K^+^ between sexes have not been fully explored. However, studies examining angiotensin II (AngII)-stimulated aldosterone secretion suggest that premenopausal women, exhibit greater sensitivity to RAAS stimulation. For example, in the Hypertensive Pathotype cohort, acute AngII infusion (3 ng/kg over 1 h) led to higher aldosterone secretion in women than in men [121]. This enhanced sensitivity was specific to premenopausal women, implicating estrogen in modulating adrenal responsiveness. These observations have been supported by in vivo and ex vivo studies in rats, which showed that females exhibit higher aldosterone secretion in response to increased extracellular K^+^ and AngII stimulation, which is explained by greater expression of aldosterone biosynthetic enzymes in female adrenals [121, 129].

While the effects of gonadal hormones on aldosterone secretion remain not fully understood, gonadectomy studies in rodents suggest that both estrogen and testosterone can suppress aldosterone production. In ovariectomized rats, estrogen depletion led to increased aldosterone levels, an effect reversed by E2 replacement [132]. E2 also inhibits AngII-stimulated aldosterone secretion in vitro [48, 106, 133], but E2 effect on K^+^-stimulated aldosterone secretion is unknown. Similarly, testosterone inhibits both basal and AngII-stimulated aldosterone secretion [134], likely by downregulating the expression of key steroidogenic enzymes [135]. Together, these data suggest that both E2 and testosterone act to limit aldosterone synthesis, raising the possibility that other factors, such as sex chromosome complement or environmental influences like diet and body volume status, may contribute to observed sex differences in aldosterone levels and responses.

An intriguing observation is that sex differences in plasma K^+^ persist into the postmenopausal and aging populations, while the sex-specific regulation of aldosterone diverges between premenopausal and postmenopausal periods, suggesting that aldosterone levels become less sensitive to K^+^ variations in the aging population, probably contributing to the higher hypokalemia risk in postmenopausal women. Indeed, adrenal responsiveness to K^+^ fluctuations requires careful characterization across the reproductive years and into older age in both sexes.

### Sex-specific regulation of intestinal K^+^ handling remains unexplored

Although to a lesser extent than the kidneys, the intestinal contribution to K^+^ balance is highly significant. When an average human body ingests 100 mEq/day of K^+^ in the diet, 90% of this amount is absorbed by the intestine [136]. In healthy humans, fecal K^+^ excretion represents < 10% of ingested K^+^, but can increase in pathologies such as hyperaldosteronism, and intestinal disorders including diarrhea, severe ulcerative colitis, and adenomas [1, 136, 137]. Additionally, when urinary K^+^ excretion is compromised, such as in end-stage renal disease, a compensatory increase in fecal K^+^ excretion is observed [138, 139]. In mammals, K^+^ is absorbed in the small intestine and to a lesser extent in the colon, by passive diffusion through a paracellular pathway, and by active transcellular transport, mediated by the apical H^+^/K^+^-ATPases [140–142], and the basolateral NKCC1 [142, 143]. K^+^ absorption is regulated by dietary intake, as evidenced by animal studies showing that K^+^ depletion increases intestinal K^+^ absorption by two-fold [144]. K^+^ secretion occurs in the colon, and is enhanced by K^+^ intake [145] and the kaliuretic hormone Aldosterone as indicated by increased fecal K^+^ secretion in hyperaldosteronism and its decrease in animals lacking Aldosterone secretion [146]. K^+^ is secreted by the enterocyte via the apical Ca [2]^+^-dependent BK channels in exchange with Na^+^, absorbed by the apical ENaC, both regulated by Aldosterone [137, 146–148]. The driving force of this process is ensured by the activity of Na^+^/K^+^-ATPase on the basal membrane [137].

Intestinal function in mammals exhibits clear sex differences as the intestine is responsive to both estrogen and testosterone [149–154], however, it is unknown whether intestinal K^+^ handling differs by sex. Given the role of estrogen in regulating renal ENaC and intracellular Ca^2+^, a key modulator of BK function [155]; in addition to the described sex differences in other intestinal ion transport such as Cl^−^ [155, 156], it is anticipated that K^+^ absorption and/or secretion might exhibit a sex-dependent pattern. For instance, it is unknown whether key transporters involved in K^+^ homeostasis, including intestinal H^+^/K^+^-ATPase, NKCC1, ENaC, and BK channels, exhibit sex-specific differences in expression or in their responses to physiological challenges such as dietary manipulation, menopause or andropause. It also remains unclear whether the intestinal epithelium differs between sexes in its sensitivity to aldosterone stimulation. Elucidating these mechanisms is crucial for developing sex-specific nutritional and therapeutic strategies, particularly in conditions in which renal K^+^ excretion is impaired.

## Clinical implications of sex-specific plasma K^+^ regulation

### Women are more susceptible to osteoporosis

Retrospective cross-sectional studies in larger cohorts showed that K^+^ intake is positively associated with Bone Mineral Density (BMD) [157, 158]. Specifically, analysis of the Korean NHANES (2008–2011) of 8,732 men and postmenopausal women over age 50, reported that higher K^+^ intake was positively associated with greater BMD in both sexes [157]. In cross-sectional and 4-year longitudinal analyses of the Framingham Heart Study survivors (n = 1164, mean age: 75y, 62% women), K^+^ intake was associated with higher BMD in both men and women [158]. Importantly, K^+^ benefits on bone health occur in young and older adults regardless of hormonal status. Indeed, a short-term (5 days) controlled feeding study involving young healthy adults (n = 13, age 21–41, 30% women), showed that K^+^ deprivation was associated with higher urinary Ca^2+^. At the same time, while K^+^ supplementation was associated with lower urinary Ca^2+^, with KHCO3 having a more robust effect than KCl [159]. In older individuals (n = 171, mean age 63, 56% women), 3 months of treatment with KHCO3 but not KCl was associated with reduced urinary Ca [2]^+^ loss and lower bone resorption [160], suggesting an additional role for acid–base balance in K^+^ benefits on bones. Urinary Ca^2+^ was reduced after four weeks of KHCO3 supplementation in both men and women over 50 [161]. K^+^-citrate supplementation helped prevent urinary Ca^2+^ loss induced by a high-salt diet in postmenopausal women (n = 52, mean age: 65) [162].

The benefits of high K^+^ intake on bone health involve renal and extrarenal mechanisms. Data from animal models show that Ca^2+^ uptake in the distal nephron occurs in the DCT2/CNT mediated by TRPV5 [163, 164], and is influenced by NCC activity [165, 166]. Due to the inhibitory effect of K^+^ on NCC [71, 89, 91], K^+^ intake likely mimics the known hypocalciuric effect of thiazides [165, 166]. Furthermore, TRPV5 activity is positively regulated by α-Klotho as shown in mouse models of α-Klotho deficiency [167, 168]. Recent data revealed that α-Klotho is highly expressed in the human and murine CNT, and dietary K^+^ supplementation in mice increases renal α-Klotho expression, with KHCO3 exerting a stronger effect than KCl [169], which parallels the observed K^+^ benefits on urinary Ca^2+^ excretion. In addition to its renal effects, extracellular K^+^ directly influences bone cell function. In murine bone cell cultures exposed to decreasing extracellular K^+^ concentrations, low K^+^ lowered intracellular pH, increased Ca^2+^ efflux and β-glucuronidase release (a marker of osteoclast activity), and suppressed collagen synthesis, all changes that favor bone resorption [170].

Based on the above evidence, one may expect that the susceptibility of women to hypokalemia predisposes them to develop lower BMD. Consistent with this assumption, data from the US NHANES 2017–2018 show osteoporosis exhibits a striking sex disparity, with postmenopausal women experiencing roughly four times the prevalence seen in age-matched men [171]. An epidemiological analysis by the International Osteoporosis Foundation across 29 European countries in 2019, estimated that 25.5 million women and 6.5 million men were affected, reflecting a nearly fourfold higher prevalence in women [172]. In China, a large cross-sectional study of approximately 20,000 adults over age 40 (57% women) also found a fourfold higher prevalence in women (20.6% vs. 5.0%) [173]. Although gonadal hormones play an undeniable role in bone homeostasis, as evidenced by the accelerated bone loss in postmenopausal women [174], a growing body of evidence suggests that the effects of K^+^ on bone physiology and Ca^2+^ regulation might be largely independent of estrogen. Despite this, the contribution of K^+^-related sex differences to women’s heightened vulnerability to osteoporosis remains poorly understood. Large-scale human cohort studies that rigorously account for key confounders, including estrogen decline, dietary intake, and body composition, would be instrumental in addressing this knowledge gap. While such studies are costly and logistically challenging, animal models, by enabling controlled manipulation and mechanistic interrogation, represent a powerful complementary approach for establishing causal links between the sex-specific K^+^ handling and osteoporosis risk.

### Women are more prone to salt-sensitive hypertension after age 50

Findings from the Third US NHANES III (~ 9,900 participants, 50% women) demonstrated that women of reproductive age exhibit lower blood pressure than age-matched men. In contrast, the menopausal transition is associated with an accelerated rise in blood pressure (BP), leading to greater susceptibility to hypertension in postmenopausal women compared to age-matched men [175]. The decline in sex hormones contributes substantially to this risk, as evidenced by increased BP following surgically induced menopause [176]; however, hormone replacement therapy does not consistently restore BP, suggesting additional regulatory mechanisms [177].

Salt-sensitive hypertension accounts for approximately half of all hypertension cases [178–180]. The Dietary Approaches to Stop Hypertension trial (412 patients, 57% women) showed that women exhibit greater salt sensitivity than men, independent of menopausal status [181]. Human epidemiologic and feeding studies further demonstrate that higher K^+^ intake not only lowers BP but also reduces salt sensitivity [181–183], while rodent studies show that K^+^ deficiency promotes salt-sensitive hypertension [184–186]. Supporting this, the GenSalt study in China (1,906 participants; 47% women) found that BP responses to both salt and K^+^ intake were significantly greater in women than in age-matched men, independent of hormonal status [187].

The molecular mechanisms underlying heightened salt-sensitive hypertension in postmenopausal women remain poorly defined, but it is more likely to involve renal and vascular mechanisms. Human and animal studies provide some insights: in the kidney, NCC expression is higher in females than in males [10, 114, 188], potentially rendering women more vulnerable to low-K^+^-induced salt retention. Moreover, postmenopausal women display higher aldosterone levels [128], whereas premenopausal women show greater sensitivity to AngII-stimulated aldosterone release [121]. Studies in rodents revealed K^+^-deficiency induces abnormal upregulation of vasoconstrictive mediators such as AngII and endothelin-1 [184], and promotes renal inflammation and injury [184, 189], though it is unclear whether these effects follow a sex-specific pattern. Additionally, extracellular K^+^ stimulates endothelium-dependent vasodilation in humans and animal models of hypertension [190–192], whereas prolonged K^+^ deficiency induces vascular calcification and arterial stiffness in mice [193]. Since menopause itself increases arterial stiffness [194, 195], K^+^ deficiency may further accelerate this process.

In summary, salt sensitivity of blood pressure is more pronounced in women, both in terms of the magnitude of BP response to dietary salt, and the overall prevalence of salt sensitivity. Women’s predisposition to hypokalemia likely contributes to their greater risk of salt-sensitive hypertension, which becomes more apparent after the loss of sex hormone protection. The underlying mechanisms probably involve an interaction of enhanced salt retention, increased renal injury, and accelerated arterial stiffness. Further studies are needed to clarify the role of K^+^ in the sex-specific pathogenesis of salt-sensitive hypertension.

### Sex-specific risk of dyskalemia-associated cardiovascular disease

Both hyperkalemia and hypokalemia can have severe consequences on cardiovascular health. The sex differences in dyskalemia risk render men and women differentially exposed to cardiovascular disease. While hypokalemia most commonly arises from chronic K^+^ imbalance or depletion, hyperkalemia develops as an acute or chronic condition with potentially life-threatening consequences. Importantly, in advanced chronic kidney disease, chronic hyperkalemia represents a major concern in the follow-up of CKD patients. Indeed, raising extracellular K^+^ reduces the resting membrane potential by making it less negative, shortens action potential duration, and impairs cardiac ion channels availability and conduction, leading to severe arrhythmia (for review [196]). Consistent with the higher male susceptibility to hyperkalemia, epidemiological studies showed that hyperkalemia-associated cardiovascular risk is higher in men than age-matched women. Indeed, in the NEFRONA cohort, hyperkalemia was associated with a higher incidence of cardiovascular events [40]. Similarly, a retrospective analysis of the Centers for Disease Control and Prevention Wide-Ranging Online Data for Epidemiologic Research (1999–2020) database showed that age-adjusted mortality rates for hyperkalemia-related cardiac arrest were consistently higher in men than in women [197]. As highlighted above, different cohorts from Spain, France and the US have shown that the majority of exercise-related sudden cardiac death victims are men [66–68].

On the other hand, women are more susceptible to hypokalemia-associated cardiac arrhythmias such as acquired long QT syndrome [198, 199]. Indeed, hypokalemia results in inhibiting cardiac repolarizing K^+^ currents, leading to a prolonged QT interval on the ECG and an increased risk of life-threatening arrhythmias such as torsades de pointes, also more common in women [200, 201]. Long QT syndrome is caused by mutations in KCNQ1 and KCNE1 genes, encoding for the pore-forming Kv7.1 α subunit and the auxiliary KCNE1β subunit, respectively, components of the I_Ks_ conducting channel, a key outward K⁺ current responsible for repolarization of the cardiac action potential [202, 203]. Interestingly, I_Ks_ channel activity is inhibited by estrogen and enhanced by androgen, resulting in the sex difference in QT interval [204, 205]. Additionally, human and animal studies showed estrogen results in vasodilation through enhancing vascular BK channel activity [206–208]. Although the sex difference in long QT syndrome is primarily attributed to normal sex-specific cardiac electrophysiology, namely longer QT interval in women than men [201, 209], lower plasma K^+^ levels in women may further exacerbate this vulnerability; however, this possibility remains to be experimentally validated.

## Conclusions, recommendations, and open questions

Sex differences in K^+^ homeostasis are observed across diverse populations worldwide, indicating that they are independent of geographic or ethnic background. Women have a higher prevalence of hypokalemia and are more susceptible to drug-induced hypokalemia, particularly with thiazide and loop diuretics and -lactam antibiotics. Accordingly, hypokalemia-associated conditions, such as osteoporosis, postmenopausal hypertension and hypokalemia-induced cardiac arrhythmias, are more common in women. In contrast, men are more prone to hyperkalemia and its consequences, including hyperkalemia-associated cardiac disease and sudden cardiac death. Although the mechanisms underlying this sexual dimorphism remain incompletely understood, available evidence implicates the kidney, skeletal muscle, aldosterone signaling, and likely the intestine. Together, these findings support revisiting nutritional recommendations and clinical management strategies to better accommodate sex-specific needs across the lifespan.

Although routine serum K^+^ monitoring after drug initiation or dose escalation is standard, additional sex-specific considerations are warranted. In women, K^+^-wasting medications should be initiated at the lowest effective dose, whereas in men, K^+^-sparing drugs should likewise be used cautiously. Combinations of multiple K^+^-wasting agents in women or multiple K^+^-sparing agents in men should be avoided. For long-term therapy, thiazide-K^+^-sparing combinations (e.g., thiazide plus amiloride) may provide a safer alternative.

## Major open questions in the field:

### Intestinal K^+^ handling

Across the lifespan, and whether intestinal K^+^ transport can be therapeutically targeted to preserve K^+^ balance during declining renal function remain unknown, yet it is critical for developing sex-specific nutritional and management strategies in chronic kidney disease.

### Pediatric K^+^ balance

Sex differences in K ^+^ homeostasis should be defined in pediatric populations, both under physiological conditions and during exposure to commonly used medications, such as β-lactam antibiotics.

### Clinical relevance and causality

Establishing causal links between sex-specific K^+^ handling and clinical outcomes, supported by mechanistic insight, is essential for the development of sex-specific therapeutic approaches.

### Renal K^+^ transport mechanisms

Defining sex differences in renal K^+^ transport is critical, especially in the context of hormone-based therapies.

### Organ prioritization, buffering versus excretion

Although both the kidney and skeletal muscle contribute to sex differences in K⁺ homeostasis, it remains unknown whether males and females differentially prioritize intracellular buffering versus renal excretion. This distinction may determine susceptibility to dyskalemia during dietary challenges, pharmacologic interventions, or disease states affecting the muscle or the kidney.

## Limitations of the observational K^+^ data

Observational studies of dietary K^+^ intake are fundamentally limited by confounding, including co-variation with overall diet quality, Na^+^ intake, comorbidities, and socioeconomic differences between men and women. Estimates derived from food frequency questionnaires or spot urine samples incompletely reflect true potassium intake and provide limited insight into whole-body K^+^ status, which integrates extracellular and intracellular pools. Although controlled feeding studies improve exposure assessment, K^+^ derived from fruits and vegetables is inseparable from other beneficial components of plant-based diets, precluding isolation of K^+^-specific, sex-dependent effects. Furthermore, most cohorts rely on single or short-term assessments, limiting evaluation of longitudinal K^+^ balance such as menstrual cycle–related K^+^ fluctuations. Consequently, observational data cannot establish causality or delineate the renal, hormonal, and extrarenal mechanisms underlying sex differences in potassium homeostasis and associated clinical outcomes.

## Data Availability

No datasets were generated or analysed during the current study.

## Abbreviations

AngII: Angiotensin II
ARH: Autosomal recessive hypercholesterolemia
BK: Flow-activated, calcium-dependent, Big-K⁺ channel
BMD: Bone mineral density
BP: Blood pressure
CD: Collecting duct
CDC: Centers for disease control and prevention
CKD: Chronic kidney disease
CNT: Connecting tubules
DCT: Distal Convoluted Tubules
E2: 17β-Estradiol
ENaC: Epithelial sodium channel
FCG: Four core genotype
HCTZ: Hydrochlorothiazide
KCC3a: Potassium and chloride cotransporter, isoform 3a
KO: Knock-out
NaPi2a: Sodium-dependent phosphate transporter, isoform 2a
NCC: Sodium and chloride cotransporter
NHE3: Sodium hydrogen exchanger
NHANES: National health and nutrition examination survey
NIH: American national institute of health
NKCC2: Sodium, potassium, and chloride cotransporter, isoform 2
PT: Proximal tubules
RAAS: Renin–angiotensin–aldosterone system
ROMK: Renal outer medullary potassium channel
SCC: Sex chromosome complement
SCREAM: Stockholm CREAtinine measurements
TAL: Thick ascending limb
TRPV5: Transient receptor potential vanilloid, isoform 5
